# Imaging of mRNA–Protein Interactions in Live Cells Using Novel mCherry Trimolecular Fluorescence Complementation Systems

**DOI:** 10.1371/journal.pone.0080851

**Published:** 2013-11-15

**Authors:** Juan Yin, Duanhao Zhu, Zhiping Zhang, Wei Wang, Jinyu Fan, Dong Men, Jiaoyu Deng, Hongping Wei, Xian-En Zhang, Zongqiang Cui

**Affiliations:** 1 State Key Laboratory of Virology, Wuhan Institute of Virology, Chinese Academy of Sciences, Wuhan, China; 2 Graduate University of Chinese Academy of Sciences, Beijing, China; University of Toronto, Canada

## Abstract

Live cell imaging of mRNA-protein interactions makes it possible to study posttranscriptional processes of cellular and viral gene expression under physiological conditions. In this study, red color mCherry-based trimolecular fluorescence complementation (TriFC) systems were constructed as new tools for visualizing mRNA–protein interaction in living cells using split mCherry fragments and HIV REV-RRE and TAT-TAR peptide-RNA interaction pairs. The new mCherry TriFC systems were successfully used to image RNA–protein interactions such as that between influenza viral protein NS1 and the 5’ UTR of influenza viral mRNAs NS, M, and NP. Upon combination of an mCherry TriFC system with a Venus TriFC system, multiple mRNA–protein interactions could be detected simultaneously in the same cells. Then, the new mCherry TriFC system was used for imaging of interactions between influenza A virus mRNAs and some of adapter proteins in cellular TAP nuclear export pathway in live cells. Adapter proteins Aly and UAP56 were found to associate with three kinds of viral mRNAs. Another adapter protein, splicing factor 9G8, only interacted with intron-containing spliced M2 mRNA. Co-immunoprecipitation assays with influenza A virus-infected cells confirmed these interactions. This study provides long-wavelength-spectrum TriFC systems as new tools for visualizing RNA–protein interactions in live cells and help to understand the nuclear export mechanism of influenza A viral mRNAs.

## Introduction

 Intracellular monitoring of RNA–protein interactions is of great importance for understanding the posttranscriptional processes of gene expression, as well as the virus–host interactions. A wide array of techniques exists to label and monitor proteins and/or RNA molecules in live cells [1-4]. However, methods for visualization of RNA–protein interactions in live cells are lacking. Current strategies include fluorescence resonance energy transfer (FRET), RNA-binding mediated FRET (RB-FRET), and trimolecular fluorescence complementation (TriFC) [5–7]. Of these, TriFC is a straightforward technique with great potential [8,9]. TriFC was first built by Rackham and Brown based on the protein complementation technique which originally developed to study protein-protein interactions in living cells [5]. Using this approach, an mRNA of interest is tagged with an *ms2* cassette while the MS2 coat protein is fused to a split fragment of a yellow fluorescent protein (YFP) variant (Venus), while the complementary portion of Venus is fused to an RNA-binding protein of interest. If the RNA-binding protein interacts with the RNA sequence of interest, the two separate Venus fragments unite, emitting a YFP signal.

Until now, there have only been a few examples of imaging RNA–protein interactions using the TriFC system. The split Venus may not be the best reporter system for TriFC because of its relative higher background than some other FC systems. In addition, the MS2 operator-MS2 coat protein interaction is not the only interaction pair which could be used to build TriFC system. Recently, other interactions between RNA aptamers and viral peptides have been used to trigger complementation of the split fluorescent protein [10,11]. In this study, we report a novel modified TriFC system based on an mCherry bimolecular fluorescence complementation (BiFC) system we developed in a previous study [12]. It exploits known RNA-protein interaction pairs such as HIV REV-RRE and HIV TAT-TAR) [13–15]. We used this system to study influenza A virus nuclear export mechanisms by investigating possible interactions between influenza A viral mRNAs and cellular adapter proteins.

Influenza viruses are segmented *Paramyxoviridae* RNA viruses that are capable of causing animal and human flu andpandemics. As is well known, a new strain of influenza A virus H7N9 is causing a lethal flu outbreak in China [16,17]. Influenza A virus is one of very few RNA viruses that synthesize mRNA in the nucleus of infected cells, and these influenza viral mRNAs must be exported from the nucleus for viral protein synthesis [18]. There are three different types of influenza virus mRNA: mRNAs that do not contain introns (PA, PB1, PB2, HA, NA, and NP mRNA), mRNAs that containing introns but that are not spliced (M1 and NS1 mRNAs), and mRNAs with introns that undergo splicing (M2 and NS2 mRNAs). It has been suggested that the nuclear export of influenza A virus mRNAs is orchestrated by several complex mechanisms [19]. Generally, mRNAs are exported via the cellular chromosome region maintenance 1 (CRM1) pathway or by the Tip-associated protein (TAP) pathway [20]. We and others have shown that influenza A virus mRNAs favor the TAP pathway [21,22]. However, the specific constituents of the TAP pathway that are required for viral mRNA nuclear export remain unclear.

A series of adapter proteins such as Aly/REF, UAP56, hnRNP A1, and 9G8 may participate in the cellular TAP export pathway [23–25], but whether viral mRNAs bind these adapter proteins is unknown. And viral mRNAs may selectively utilize the cellular nuclear export factors and simultaneously inhibit cellular mRNA export [26,27]. It is still far away from clarifying the mechanism of the nuclear export of influenza A viral mRNAs. By using the mCherry TriFC system, we visualized interactions between different influenza A viral mRNA molecules and some adapter proteins of the TAP pathway in live cells. These results will help elucidate the nuclear export mechanism of influenza viral mRNAs.

## Results

### Design and construction of the mCherry TriFC system

**Figure 1:**
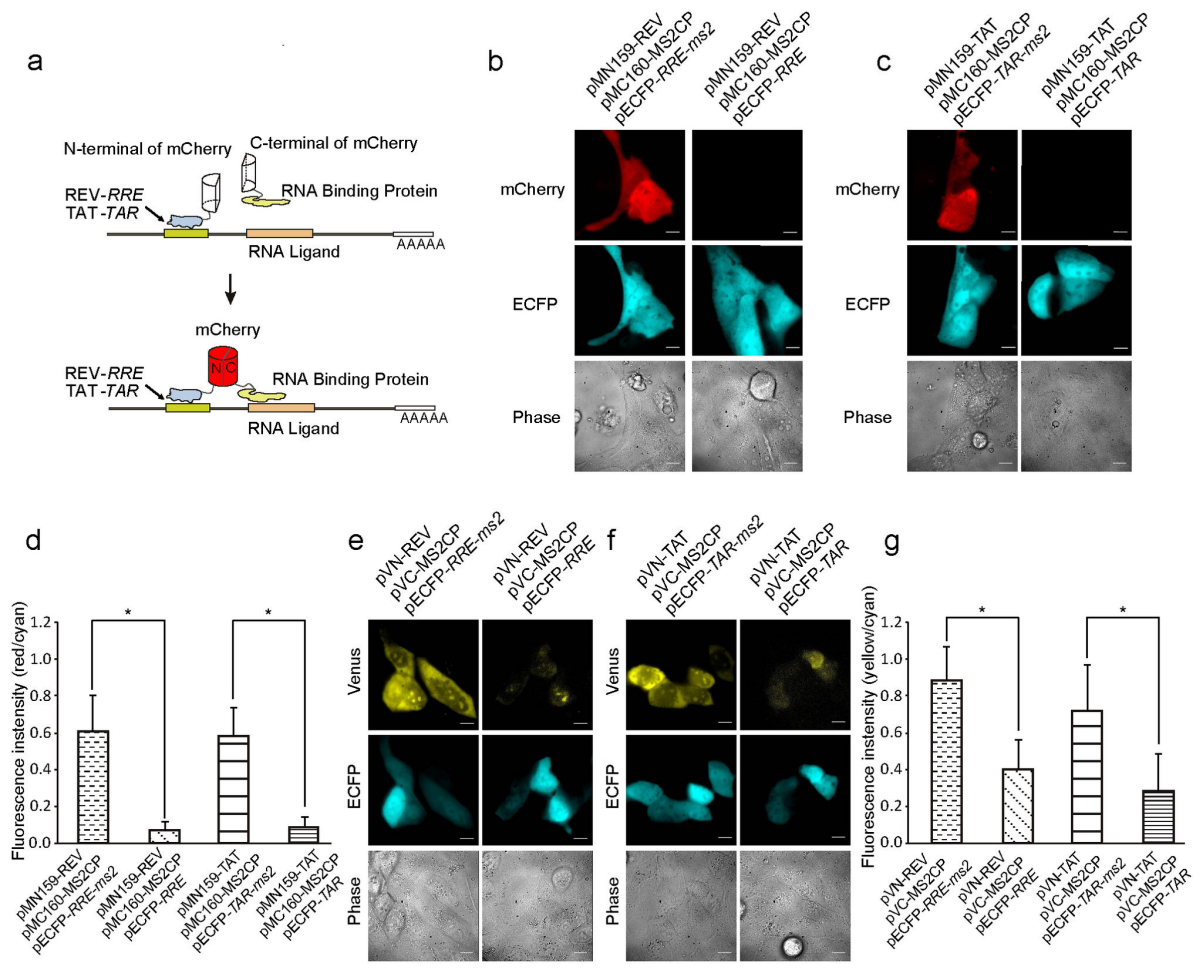
Construction of the TriFC systems. (a) Schematic illustration of the TriFC constructs composed of split mCherry fragments, HIV Rev-*RRE*, and Tat-*TAR* peptide-RNA pairs. The N-terminus of mCherry (MN159) was tethered to an mRNA of interest via an HIV REV-RRE or TAT-TAR interaction, while the C-terminus of mCherry (MC160) was fused to a candidate RNA-binding protein. If the RNA-binding protein associated with the RNA sequence of interest, the two mCherry fragments were united and a red fluorescent protein (RFP) signal was emitted. (b) The mCherry-*RRE*-TriFC system detects the interaction between bacteriophage MS2 coat protein (MS2CP) and its RNA operator *ms2*. No RFP signal was detected when *ms2* was absent. The ECFP signal was an internal control demonstrating that pECFP-*RRE*-*ms2* or pECFP-*RRE* were expressed (c) The mCherry-*TAR*-TriFC system detects the MS2CP-*ms2* interaction. No RFP signal was detected when *ms2* was absent (d) Quantitative analysis of the signals detected in (1b) and (1c) based on the fluorescence intensity ratio TriFC/ECFP (red/cyan). Quantitative analysis data are presented as mean values + S.D. (n = 30). (f) The Venus-*RRE*-TriFC system detects the MS2CP-*ms2* interaction. When the *ms2* was absent, a background YFP signal was detected. (g) The Venus *TAR*-TriFC system detects the MS2CP-*ms2* interaction. When *ms2* was absent, a slightly background YFP signal was detected. (h) Quantitative analysis of the TriFC signals in (1f) and (1g) based on the fluorescence intensity ratio of TriFC/ECFP (yellow/cyan). Quantitative analysis data are presented as mean values + S.D. (n = 30). The statistical significance was evaluated using a Student’s t-test. * indicates *P*-values <0.01. Bar, 10 µm.

 The mCherry red fluorescent protein (RFP) was used as the fluorescence reporter in the TriFC system for this study. The N-terminus of mCherry (MN159) was tethered to an mRNA of interest by HIV REV-RRE or TAT-TAR interaction (Figure 1a). The C-terminus of mCherry (MC160) was fused to an RNA-binding protein candidate. *RRE* or *TAR* sequences, along with any RNA sequence of interest, were ligated into the backbone plasmid pECFP-C1. If the RNA-binding protein was able to associate with the RNA sequence of interest, it united the two mCherry termini and yielded an RFP signal. Using this approach, two sets of mCherry TriFC plasmids were constructed. The mCherry-*RRE*-TriFC system comprised plasmids pMN159-REV, pMC160 and pECFP-*RRE*, while the mCherry-*TAR*-TriFC system comprised plasmids pMN159-TAT, pMC160, and pECFP-*TAR*.

 The well-known interaction of bacteriophage MS2 coat protein and its RNA operator was used to test the new TriFC systems. The operator *ms2* and MS2 coat protein coding sequences were incorporated into TriFC plasmid vectors of mCherry-*RRE*-TriFC system (formed as pMN159-REV, pMC160-MS2CP and pECFP-*RRE*-*ms2* shown in Table S1)and mCherry-*TAR*-TriFC system (plasmids pMN159-TAT, pMC160-MS2CP, pECFP-*TAR*-*ms2*) and cotransfected them into MDCK cells respectively. When *ms2* RNA and the MS2 coat protein were co-expressed in the TriFC system, both mCherry-*RRE*-triFC and mCherry-*TAR*-TriFC systems could produce a bright red fluorescent signal (Figure 1b and 1c). The mCherry TriFC signal was evenly distributed throughout the cells, showing that the *ms2*-MS2 interaction has no specific subcellular localization, and that the mCherry TriFC system was capable of expressing proteins throughout the whole cell. The Enhanced Cyan Fluorescent Protein (ECFP) fluorescence signal was used as an inner control and was constantly expressed, confirming that the RNA sequences of interest (*RRE* and *TAR*) were rightly expressed. As negative controls, cells co-transfected with plasmids that did not contain *ms2* RNA or MS2 coat protein sequences did not emit an RFP signal (Figure 1b and 1c). Quantitative analysis was performed by measuring the fluorescence intensity ratio of TriFC/ECFP (red/cyan). The mCherry TriFC signals from the *ms2*-MS2 interaction were significantly higher than signals when there was no *ms2* RNA (Figure 1d). In addition, whenever any of the binding elements REV-RRE or TAT-TAR were absent, no RFP signal could be detected (data not shown).

 By using the HIV REV-RRE or TAT-TAR interaction, new Venus-based TriFC systems were also constructed in this study as alternative systems for that built by Rackham and Brown [5]. Using the same approach outlined in Figure 1a, two Venus fragments were substituted for mCherry. These two new sets of constructs were named the Venus-*RRE*-TriFC and Venus-*TAR*-TriFC systems. When *ms2* RNA and the MS2 coat protein were co-expressed using the Venus TriFC system, both Venus-*RRE*-TriFC and Venus-*TAR*-TriFC produced a yellow fluorescent protein (YFP) signal (Figure 1e and 1f). However, in control co-transfections in which there was no *ms2* RNA or MS2 coat protein, a background YFP signal could be detected (Figure 1e and 1f). Despite the presence of this background signal, quantitative analysis of the fluorescence intensity ratio TriFC/ECFP (yellow/cyan) showed that the Venus TriFC signal of the *ms2*-MS2 interaction was markedly higher than the signal emitted when there was no *ms2* RNA (Figure 1g).

### Validation of the New Constructed mCherry TriFC System

**Figure 2:**
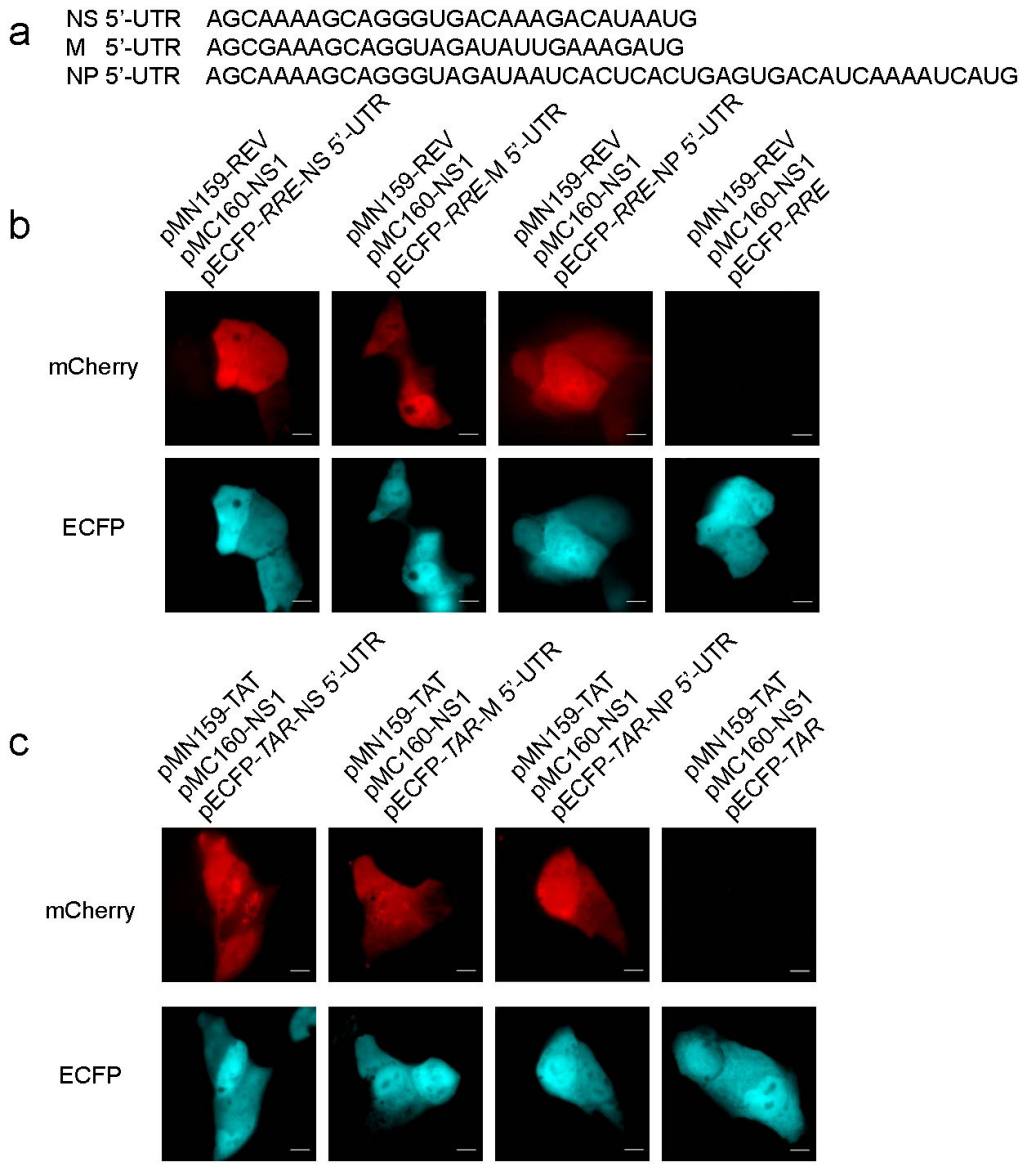
mCherry TriFC systems detect interactions between influenza viral NS1 protein and the 5’ UTR of influenza viral mRNAs NS, M, and NP. (a) Sequences of 5’ UTR of influenza viral mRNAs NS, M, and NP. (b) The mCherry-*RRE*-TriFC system co-expressing NS1 protein and the 5’ UTR of influenza viral mRNAs NS, M , and NP in MDKC cells (c) The mCherry-*TAR*-TriFC system co-expressing NS1 protein and the 5’ UTR of influenza viral mRNAs NS, M, and NP. Bar, 10 µm.

We used known mRNA–protein interactions to assess the efficacy of our mCherry TriFC system. These included interactions between the influenza viral NS1 protein and the 5’ untranslated region (UTR) of influenza viral mRNAs NS, M, and NP [28]. The 5’ UTR of influenza viral mRNAs for NS, M, and NP, as well as the NS1 gene, were separately cloned into TriFC plasmid vectors (Figure 2a and Table S1), then co-transfected into MDCK cells. As expected, when the NS1 protein and any of the 5’ UTR of influenza viral NS, M, and NP mRNAs were co-expressed, both mCherry-*RRE*-TriFC and mCherry-*TAR*-TriFC produced RFP signals (Figure 2b and 2c). The internal control (ECFP) was also expressed. Conversely,when there was no NS1 protein or any of the 5’ UTR of NP, NS, and M mRNAs, no mCherry fluorescent signal could be detected. These results verified that the new constructed mCherry TriFC system could be used to detect mRNA–protein interactions in live cells.

**Figure 3:**
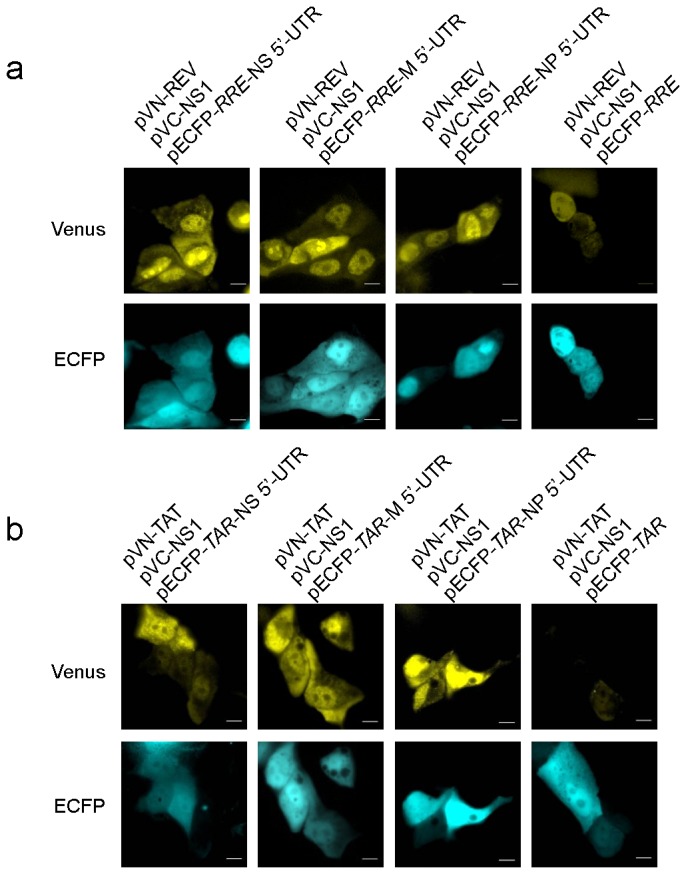
Venus TriFC systems detect interactions between influenza viral NS1 protein and the 5’ UTR of influenza viral mRNAs NS, M, and NP. (a) The Venus-*RRE*-TriFC system co-expressing NS1 protein and the 5’ UTR of influenza viral mRNAs NS, M, and NP. (b) The Venus *TAR*-TriFC system co-expressing NS1 protein and the 5’ UTR of influenza viral mRNAs NS, M, and NP. Bar, 10 µm.

By using the interactions between influenza viral NS1 protein and the 5’ UTR of viral NS, M, and NP mRNAs, the new Venus-based TriFC systems were also tested to be used for detection of mRNA–protein interactions. 5’ UTR of influenza viral mRNAs NS, M, and NP, as well as the *NS1* gene, were cloned into Venus-based TriFC plasmid vectors (Table S1) and co-transfected into MDCK cells. Live cell imaging from these co-transfections is shown in Figure 3. When the NS1 protein and any of the 5’ UTR of influenza viral mRNAs NS, M, and NP were co-expressed, both Venus-*RRE*-TriFC and Venus-*TAR*-TriFC could produce YFP signals (Figure 3a and 3b). However, negative controls also emitted a slightly background YFP signal. Nevertheless, these results further demonstrated that HIV REV-RRE and TAT-TAR pairs are suitable for construction of the new TriFC system. Considering that the lower background of mCherry-based TriFC systems than Venus systems, it is thought that the mCherry TriFC systems should be good systems for imaging mRNA–protein interactions in live cells.

### Simultaneous visualization of multiple protein-mRNA interactions by combining the mCherry TriFC and Venus TriFC systems

**Figure 4:**
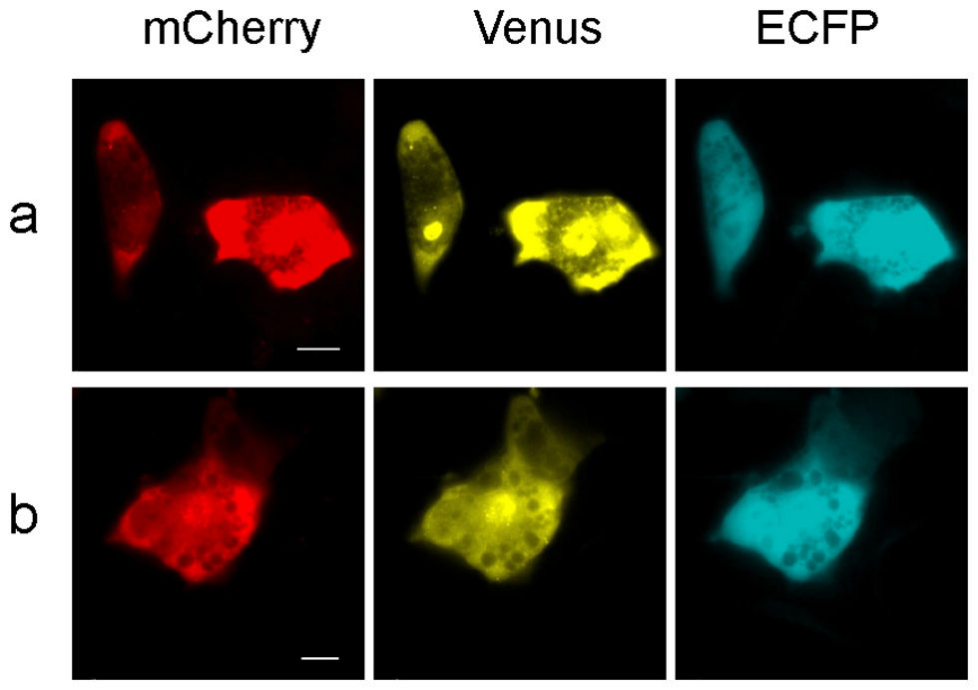
Visualization of multiple protein-mRNA interactions with combined mCherry TriFC and Venus TriFC systems in the same cells. (a) The MS2CP-*ms2* interaction detected with the mCherry-*RRE*-TriFC system and the NS1-5’ NS UTR interaction detected with the Venus-*TAR*-TriFC system in the same MDCK cells. (b) The NS1-5’ NS UTR interaction detected with the mCherry-*RRE*-TriFC system and the MS2CP-*ms2* interaction detected with the Venus-*TAR*-TriFC system in the same MDCK cells. (ECFP signal; internal control). Bar, 10 µm.

 We visualized multiple mRNA-protein interactions by combining the mCherry TriFC and Venus TriFC systems in the same cells. To do so, the mRNA–protein interaction pair MS2CP-*ms2* was incorporated into mCherry-*RRE*-TriFC vectors and the NS1-5’ NS UTR interaction pair was incorporated into Venus-*TAR*-TriFC vectors before they were co-transfected into MDCK cells. Both RFP and YFP signals were detected after co-transfection in the same cells (Figure 4a). Simultaneous visualization of both signals was also observed with the mCherry-*TAR*-TriFC vector and the Venus-*RRE*-TriFC vector (Figure 4b). These data demonstrated that the combined use of mCherry and Venus TriFC systems could simultaneously monitor interactions between two pairs of mRNA-protein interactions in the same cells.

### Cellular adapter proteins Aly and UAP56 were found to interact with influenza A viral mRNAs by using the mCherry TriFC system

**Figure 5:**
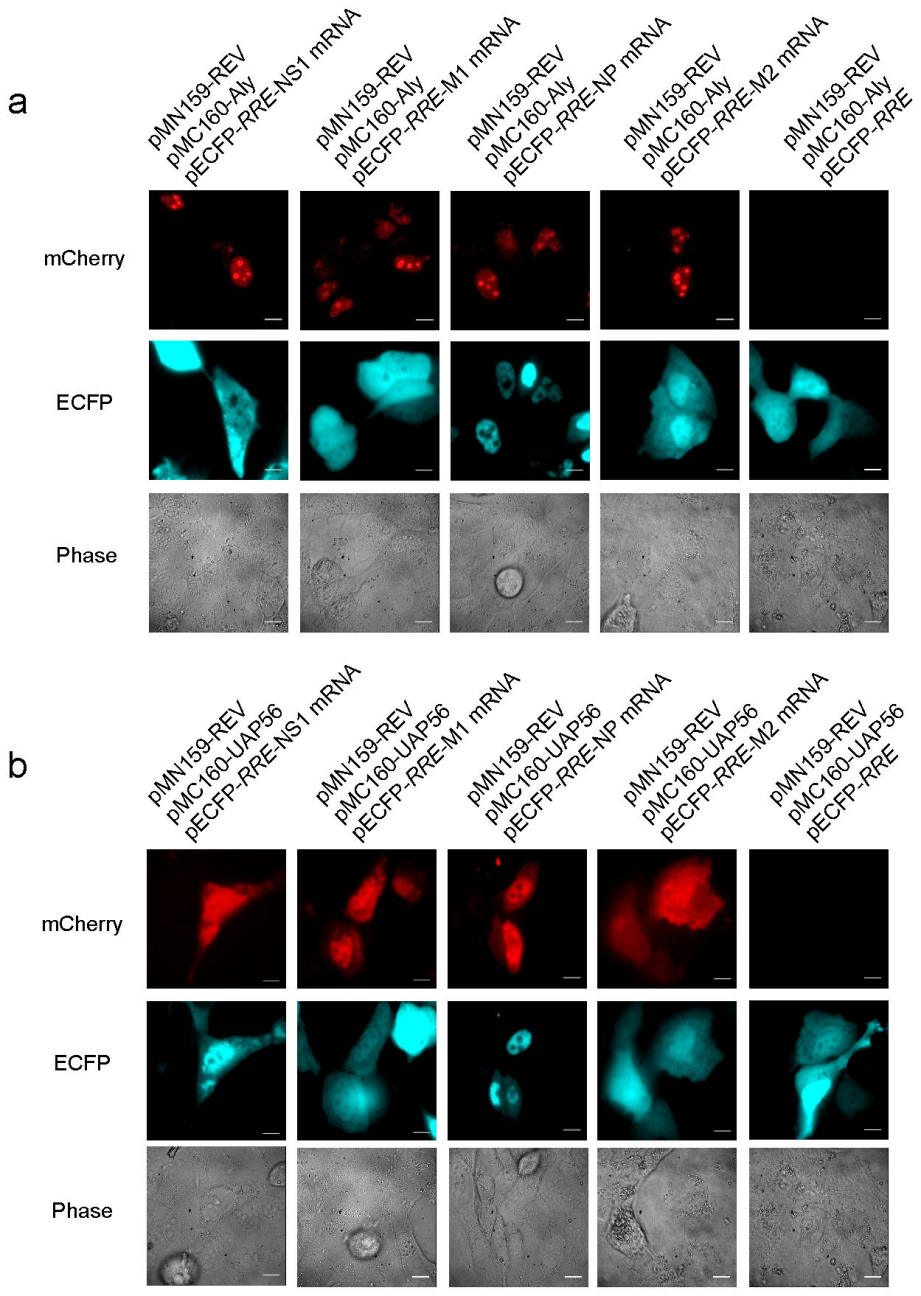
The mCherry-*RRE* TriFC system detects interactions between influenza A viral mRNAs and cellular adapter proteins Aly and UAP56. (a) Images acquired from Aly interactions with influenza A viral mRNAs. (b) Images acquired fromUAP56 interactions with influenza A viral mRNAs Bar, 10 µm.

 It is hypothesized that influenza A mRNAs are exported out of the cellular nucleus via the TAP pathway. Cellular Aly and UAP56 proteins are key adapter proteins that bind mRNAs in TAP-dependent mRNA nuclear export. We tested whether influenza A viral mRNAs bound these adapter proteins in live cells using the mCherry TriFC system. NS1 and NP mRNA (do not contain introns), M1 mRNA (intron-containing but unspliced), and M2 mRNA (produced by splicing) were chosen as viral mRNA candidates. RFP interaction signals were detected for influenza viral mRNAs NS1, M1, NP, M2 mRNAs and the adapter proteins Aly and Uap56 (Figure 5a and 5b). No RFP signal was detected for negative control co-transfections that did not include influenza viral mRNAs. These results indicated that the Aly and UAP56 adapter proteins were able to bind influenza viral mRNAs. In addition, the RFP signals between Aly and the influenza viral mRNAs were mainly localized to the nucleus, and the signal intensified in the nucleolus (Figure 5). In contrast, signals indicating interactions between UAP56 and influenza viral mRNAs were detected in the nucleus and in the cytoplasm.

### Interaction between the 9G8 adapter protein and viral M2 mRNA

**Figure 6:**
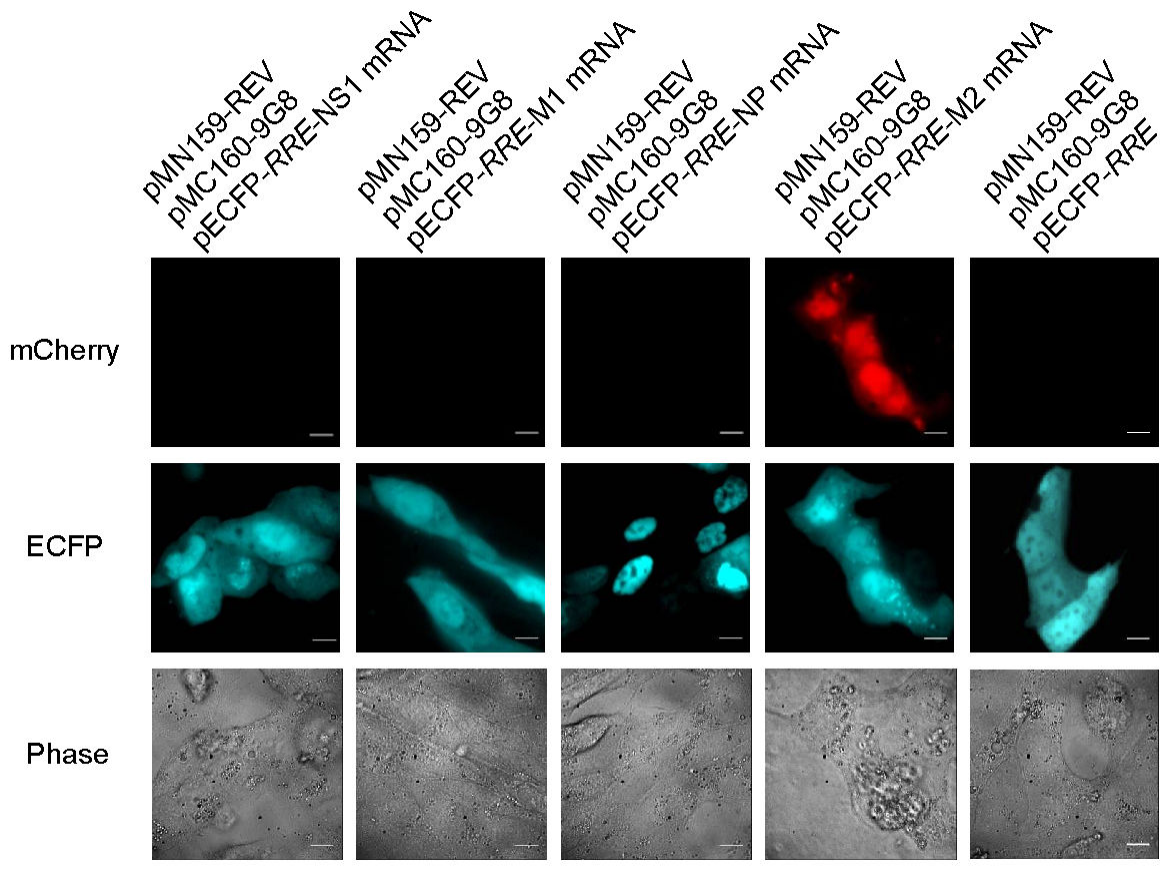
The mCherry-*RRE* TriFC system detects interactions between the protein 9G8 and influenza A viral mRNAs. Bar, 10 µm.

 We also tested whether influenza A viral mRNAs could interact with 9G8, another adapter protein and splicing factor. To do so, the 9G8 open reading frame (ORF) and influenza A viral mRNA sequences NS1, M1, NP, and M2 were cloned into the mCherry TriFC system and transfected into MDCK cells. Interestingly, RFP signals were detected between 9G8 and M2 mRNA, but not with NS1, M1, or NP mRNAs (Figure 6). These results suggested that 9G8 was able to interact with M2 mRNA upon splicing, but did not interact with other type of influenza A viral mRNAs (NS1, M1, and NP mRNAs).

### Confirmation of in vivo interactions using co-immunoprecipitation assays

**Figure 7:**
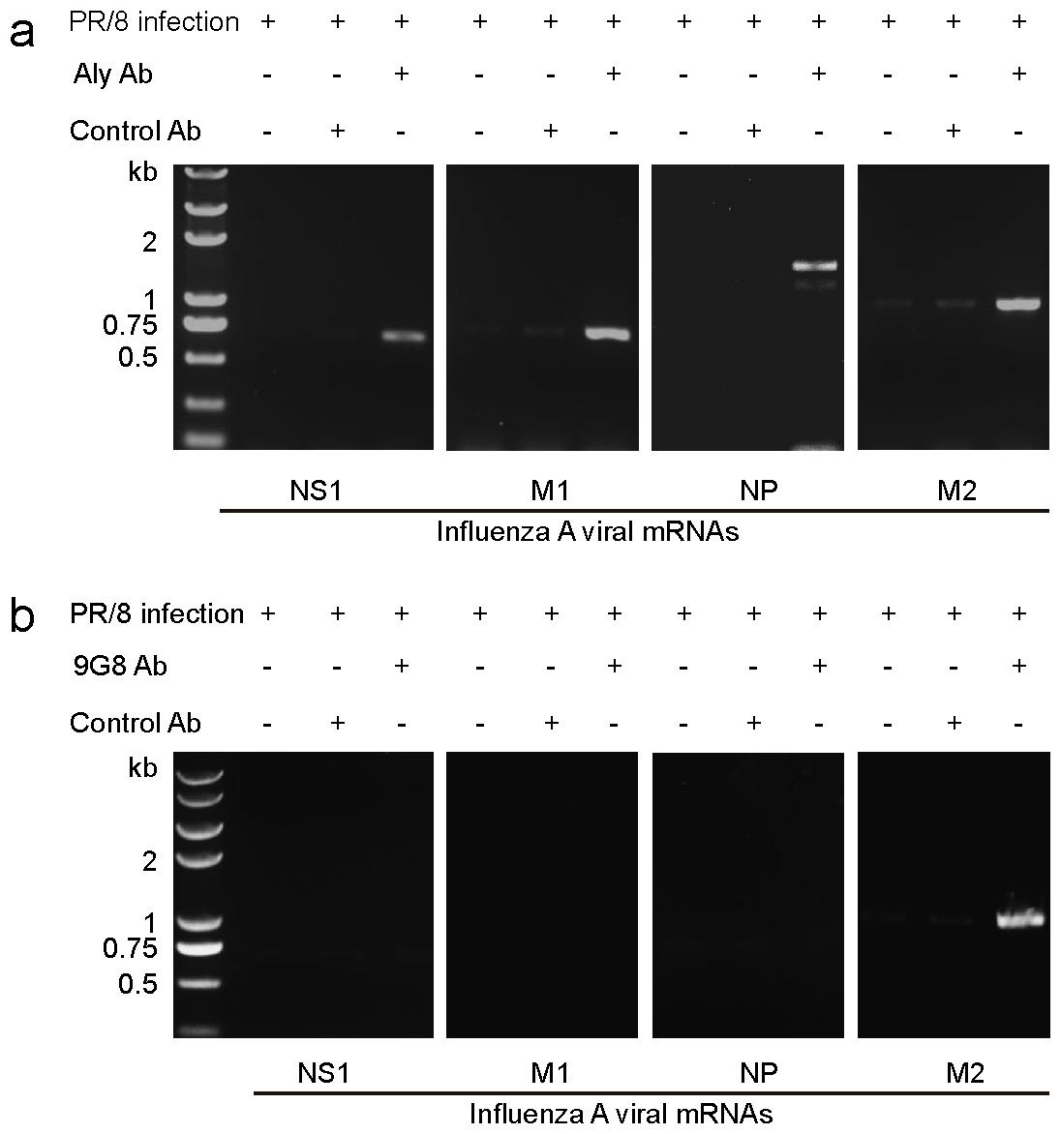
Co-immunoprecipitation assays with cellular adapter proteins. (a) Aly with influenza A viral mRNAs (b) 9G8 with influenza A virus mRNAs. Co-immunoprecipitation assays were performed with cell lysates from influenza A virus-infected MDCK cells, anti-Aly, and anti-9G8 antibodies. The control antibody was anti-GFP. RNA extracted from the immunoprecipitated samples was used for RT-PCR and agarose gel electrophoresis analyses of the presence of influenza A viral NS1, M1, NP, M2 mRNAs.

 Co-immunoprecipitation assays were performed to verify interactions detected between influenza A virus mRNAs and the adapter proteins in the TAP-dependent mRNA nuclear export pathway during viral infection. For these analyses, cell lysates prepared from influenza A virus-infected MDCK cells at 5 h post-infection (hpi) were immunoprecipitated using specific antibodies. To detect the presence of influenza A virus mRNAs NS1, M1, NP, and M2, RNA was extracted from the immunoprecipitated samples and subjected to RT-PCR and agarose gel electrophoresis. Immunoprecipitation experiments for mRNA-Aly complexes were performed with an anti-Aly antibody (Figure 7a). Influenza A virus mRNAs NS1, M1, NP, M2 mRNAs were pulled down with the anti-Aly antibody but not with the anti-GFP antibody (negative control). Similarly, no influenza A virus mRNA could be detected in non-infected cell co-immunoprecipitation samples pulled down with the anti-Aly antibody. When an anti-9G8 antibody was used, only M2 mRNA could be detected (Figure 7b). Thus, co-immunoprecipitation confirmed the interactions detected by TriFC imaging analysis.

## Discussion

 Live cell imaging can provide information about RNA–protein interactions under physiological conditions. TriFC is a good system for visualizing the RNA–protein interactions in living cells. In this study, a kind of red color TriFC system was built by using the mCherry fluorescence complement fragments and the HIV REV-RRE and HIV TAT-TAR interaction pairs of peptide-RNA. Considering good properties of mCherry such as brilliant redness, short maturation time, and the long excitation and emission wavelengths (587/610 nm), the new mCherry TriFC system would be excellent tool for analyzing RNA–protein interactions in living cells. The results also showed that the HIV REV-RRE and HIV TAT-TAR interaction pairs are good substitutes for the commonly used MS2 coat protein and its interaction with the RNA operator *ms2*, as well as other interaction pairs used in similar TriFC systems. Furthermore, a mCherry TriFC system combined with a Venus TriFC system allows for simultaneous examination of two pairs of mRNA–protein interactions in the same cells.

 We also concede that there are limitations to using the mCherry TriFC system. These include low temperature requirements for the reconstruction of split mCherry fragments, and the system’s applicability to studying the dynamic binding processes that characterize RNA-protein interactions. Further studies aimed at improving this technique are required.

 We used the mCherry TriFC system to test for interactions between influenza A viral mRNA and cellular adapter proteins needed for mRNA nuclear export. Previous studies have indicated that influenza A virus mRNAs are exported from the nucleus by the cellular TAP pathway. To facilitate passage through nuclear pores, adapter proteins act as bridges between mRNA molecules and the TAP receptor protein [29]. Aly, RNA helicase UAP56, and some splicing factors are known key adapter proteins involved in this process [30,31]. Our results show that Aly and Uap56 interact with influenza A viral mRNAs NS1, M1, NP, and M2. This suggests that these two adapter proteins are capable of directly binding three different types of influenza A viral mRNAs including intron-containing unspliced NS1 and M1 mRNA, intronless and unspliced NP mRNA, and intron-containing spliced M2 mRNA.

 Previous reports have shown that reduced UAP56 levels strongly inhibit trafficking and translation of M1, M2, NS1 viral mRNAs [32]. Our results are in accordance with this data, as they suggest that viral mRNA nuclear export is mediated via specific adapter proteins. It was also reported that siRNA silencing of Aly and UAP56 affects nuclear export of viral mRNAs, and that these two adapter proteins are recruited together to the spliced mRNA-protein complex [31,32]. However, images acquired in this study show different intracellular localization for Aly-viral mRNA interactions (nucleus and nucleolus) and UAP56-viral mRNA interactions (nucleus and cytoplasm). This suggests that Aly and UAP56 act differently in specific subcellular regions during viral mRNA processing and nuclear export.

 In this study, 9G8 only interacted with influenza A intron-containing and spliced M2 mRNA. 9G8 was originally identified as a splicing factor before it was characterized as an adapter protein that recruits mRNA in the TAP pathway [29]. The interaction between 9G8 and M2 mRNA indicates that 9G8 only participates in splicing and transport of intron-containing spliced mRNA molecules, and has no direct relationship with other types of viral mRNA molecules. These data support the notion that different types of influenza A virus mRNA molecules undergo different posttranscriptional processing mechanisms [19]. In addition, our results suggest that TAP pathway-related proteins have different roles during nuclear export of viral mRNA. As such, spliced and unspliced viral mRNAs show differential dependence on specific TAP pathway-related proteins.

 This study implies that complex mechanisms exist for the nuclear export of influenza A virus mRNAs. It may be that some TAP pathway-related proteins participate in mRNA nuclear export by directly associating with several different types of viral mRNAs, while others are more specific. This study and previous work show that virus-encoded proteins such as NS1 participate in influenza A viral mRNA nucleus export [33]. Interactions between viral proteins and TAP pathway-related proteins [34,35], may also play roles in the nuclear export of influenza A mRNAs. Nonetheless, the exact processes involved in nuclear export of influenza A mRNAs via the TAP pathway need further clarification.

 In conclusion, we developed a red TriFC system that uses a split mCherry reporter and HIV Rev-*RRE* and Tat-*TAR* peptide-RNA pairs to image RNA-protein interactions in live cells. We detected interactions between influenza A virus mRNAs and some of the key adapter proteins for mRNA nuclear export in the TAP cellular pathway. The study provides a new tool for visualizing RNA–protein interactions in live cells and helps elucidate the mechanisms underlying nuclear export of influenza A viral mRNAs.

## Materials and Methods

### Construction of the TriFC systems

 The mCherry-*RRE*-TriFC system included three plasmids: pMN159-REV, pMC160, and pECFP-*RRE*. To construct pMN159-REV, the *REV* gene was synthesized (Sangon Biotech, Shanghai, China) and cloned into pMN159 using *Eco*RI and *Sal*I. pMN159 and pMC160 were constructed in our laboratory as previously described [12]. To obtain pECFP-*RRE*, the *RRE* gene was synthesized and cloned into pECFP-C1 using *Bgl*II and *Hind*III. The mCherry-*TAR*-TriFC system included the following plasmids: pMN159-TAT, pMC160, and pECFP-*TAR*. The *TAT* gene was synthesized and cloned into pMN159 using *Eco*RI and *Sal*I. To obtain pMN159-*TAT*, the *TAR* gene was synthesized and cloned into pECFP-C1 using *Bgl*II and *Hind*III.

 The Venus-*RRE*-TriFC system included three plasmids: pVN-REV, pVC-C1, and pECFP-*RRE*. To construct pVN-REV, the *REV* gene was synthesized (Sangon Biotech, Shanghai, China) and cloned into pVN-C1 using *Eco*RI and *Sal*I. Both pVN-C1 and pVC-C1 were constructed in our laboratory as previously described [12]. The Venus-*TAR*-TriFC system included three plasmids: pVN-TAT, pVC, and pECFP-*RRE*. To obtain pVN-*TAT*, the *TAT* gene was synthesized and cloned into pVN-C1 using *Eco*RI and *Sal*I.

To construct pMC160-MS2CP, the MS2 coat protein gene (*MS2CP*) was amplified from pG14-MS2-GFP (kindly provided by Dr. Robert Singer, Yeshiva University, New York, USA) and then cloned into pMC160 using *Hind*III and *Pst*I. To obtain pECFP-*RRE*-*ms2* and pECFP-*TAR*-*ms2*, the *ms2* sequence was synthesized (Sangon Biotech, Shanghai, China) and cloned into pECFP-*RRE* and pECFP-*TAR*, respectively, using *Kpn*I and *Bam*HI. To obtain pVC-MS2CP, the *MS2CP* gene was cloned into pVC-C1 using *Hind*III and *Pst*I sites.

The *NS1* ORF was amplified by PCR and inserted into pMC160 or pVC-C1 to yield pMC160-NS1 and pVC-NS1 using *Eco*RI and *Kpn*I. The 5'UTR of M, NP, or NS mRNA were synthesized (Sangon Biotech, Shanghai, China) and cloned into pECFP-*RRE* or pECFP-*TAR* at *Kpn*I and *Bam*HI sites to obtain pECFP-RRE-M 5'UTR, pECFP-RRE-NP 5'UTR, pECFP-RRE-NS 5'UTR, pECFP-TAR-M 5'UTR, pECFP-TAR-NP 5'UTR, and pECFP-TAR-NS 5'UTR.

### Plasmids construction for interactions between influenza A mRNAs and cellular proteins

 The *Aly, UAP56*, and *9G8* ORFs were amplified by PCR from plasmids GST-UAP56, GST-Aly (kindly provided by Dr. Robin Reed, Harvard Medical School, Boston, USA) and pEGFP-C1-9G8 respectively, and cloned into pMC160 to yield pMC160-9G8, pMC160-UAP56, and pMC160-Aly. *M1*, *NP*, *M2*, and *NS1* ORFs were amplified by PCR from pGFPM703, pMALNP (kindly provided by Dr. Paul Digard, University of Cambridge, Cambridge, UK), pHW197-M (kindly provided by Dr. Richard Webby, University of Tennessee, Memphis, USA) and pEGFP-C1-NS1 respectively, and cloned into *pECFP*-*RRE* to yield pECFP-*RRE*-NS1, pECFP-*RRE*-M1, pECFP-RRE-NP, and pECFP-*RRE*-M2.

### Cell culture, transfection, and virus infection

 MDCK cells were maintained in a humidified incubator at 37 °C with 5% CO_2_ and grown in RPM1640 medium supplemented with 10% FBS, 100 U/ml penicillin, and 100 mg/ml streptomycin. Cells were plated in 35 mm tissue culture dishes at 70–80% confluence 1 day before transfection. The cells were transfected with plasmids using Lipofectamine 2000 reagent (Invitrogen, USA) according to the manufacturer’s instructions. Transfected cells were incubated at 37 °C (5% CO_2_) for 16−18 h, followed by 1–2 h at 4 °C or at 25 °C overnight (5% CO_2_) before imaging analyses. Cells were observed using inverted fluorescence microscopy after replacing the medium with fresh medium.

 For virus infections, MDCK cells were infected with the influenza A virus strain A/PR/8/34. The virus was added to cells at a MOI of 10–100 and adsorbed for 60 min at 37 °C. After removing the virus dilution, cells were maintained in infecting media (RPMI 1640, 4 µg/ml trypsin) at 37 °C (5% CO_2_).

### Fluorescence microscopy, image acquisition and data analysis

 Cell imaging was performed with an inverted wide-field fluorescent microscope (Axiovert 200, Carl Zeiss, Germany). For ECFP signals, imaging conditions included a 438/24 nm excitation filter, 510 nm dichroic beam splitter, and 483/32 nm emission to view the ECFP signal. For mCherry signals, the imaging parameters were an 531/40 nm excitation filter, a 600 nm dichroic beam splitter, and a 593/40 emission filter. For Venus signals, the imaging parameters included a 500/24 nm excitation filter, a dichroic 515 nm beam splitter, and a 542/27 nm emission filter (all filters were obtained from Semrock, USA). Images from the mCherry, ECFP, and Venus channels were captured using a cooled CCD camera (Model Cascade 512B, Photometrics, USA) with equal exposure times. The camera was operated at 5 MHz with 16-bit digitization using MetaMorph 6.0 software (Molecular Devices, USA). The fluorescence intensity ratios of TriFC/ECFP (red/cyan) were quantified for every cell showing both red and cyan fluorescence signals. Statistical analyses were performed using the SPSS14.0 software (SPSS Inc., Chicago, IL, USA) for Windows. All *p*-values < 0.05 were considered statistically significant.

### Co-immunoprecipitation assays

Cell lysates of virus-infected MDCK cells collected at 5 h pi were used for co-immunoprecipitation assays with specific antibodies. The RNA present in the immunoprecipitates was extracted as described previously [36]. Isolated RNA pellets were resuspended in nuclease-free water and analyzed with the One Step RT–PCR kit (TaKaRa Biotech, Japan) using primers specific for influenza A virus NS1, M1, NP, M2 mRNA. Primer sequences used were the following: NS1-sense: 5' ATGGATCCAAACACTG 3'; NS1-antisense: 5' TCAAACTTCTGACCTA 3'; M1-sense: 5' ATGAGTCTTCTAACCG 3'; M1- antisense: 5' TCACTTGAACCGTTGC 3'; NP-sense: 5' ATGGCGTCCCAAGGCA 3'; NP-antisense: 5' TTAATTGTCGTACTCC 3'; M2-sense: 5' ATGAGTCTTCTAACCG 3'; M2-antisense: 5' TTACTCCAGCTCTATG 3'.

## Supporting Information

Table S1 **Plasmids used for the TriFC systems developed in this study.**
(DOCX)
