# Supplementary material for: Imaging of mRNA–Protein Interactions in Live Cells Using Novel mCherry Trimolecular Fluorescence Complementation Systems
Source: PLoS One. 2013 Nov 15;8(11):e80851. doi: 10.1371/journal.pone.0080851 (PMC3829953; doi:10.1371/journal.pone.0080851)
Supplement: Table S1 — Plasmids used for the TriFC systems developed in this study. (DOCX) [file pone.0080851.s001.docx]

**Table S1. Plasmids used for the TriFC systems developed in this study.**

| **Plasmids** | **Characterisitics** |
| --- | --- |
| pMC160 | Express the C-terminal of mCherry (MC160) |
| pMN159-REV | Express the fusion protein MN159-REV |
| pECFP-*RRE* | Express the RNA sequence of RRE and the cyan fluorescent protein |
| pMN159-TAT | Express the fusion protein MN159-TAT |
| pECFP-*TAR* | Express the RNA sequence of TAR and the cyan fluorescent protein |
| pVC | Express the C-terminal of Venus |
| pVN-REV | Express fusion protein VN-REV |
| pVN-TAT | Express the fusion protein VN-TAT |
| pMC160-MS2CP | Express the fusion protein MC160-MS2CP |
| pECFP-*RRE*-*ms2* | Express the RNA sequence of RRE-ms2 and the cyan fluorescent protein |
| pECFP-*TAR*-*ms2* | Express the RNA sequence of TAR-ms2 and the cyan fluorescent protein |
| pECFP-*RRE*-M 5'UTR | Express the RNA sequence of RRE-M 5'UTR and the cyan fluorescent protein |
| pECFP-*RRE*-NP 5'UTR | Express the RNA sequence of RRE-NP 5'UTR and the cyan fluorescent protein |
| pECFP-*RRE*-NS 5'UTR | Express the RNA sequence of RRE-NS 5'UTR and the cyan fluorescent protein |
| pECFP-*TAR*-M 5'UTR | Express the RNA sequence of TAR-M 5'UTR and the cyan fluorescent protein |
| pECFP-*TAR*-NP 5'UTR | Express the RNA sequence of TAR-NP 5'UTR and the cyan fluorescent protein |
| pECFP-*TAR*-NS 5'UTR | Express the RNA sequence of TAR-NS 5'UTR and the cyan fluorescent protein |
| pMC160-NS1 | Express the fusion protein MC160-NS1 |
| pVC-NS1 | Express the fusion protein VC-NS1 |
| pMC160-Aly | Express the fusion protein MC160-ALY |
| pMC160-UAP56 | Express the fusion protein MC160-UAP56 |
| pMC160-9G8 | Express the fusion protein MC160-9G8 |
| pECFP-*RRE*-NS1 | Express the RNA sequence of RRE-NS1 and the cyan fluorescent protein |
| pECFP-*RRE*-M1 | Express the RNA sequence of RRE-M1 and the cyan fluorescent protein |
| pECFP*-RRE*-NP | Express the RNA sequence of RRE-NP and the cyan fluorescent protein |
| pECFP-*RRE*-M2 | Express the RNA sequence of RRE-M2 and the cyan fluorescent protein |
